# The Delivery of mRNA Vaccines for Therapeutics

**DOI:** 10.3390/life12081254

**Published:** 2022-08-17

**Authors:** Nitika, Jiao Wei, Ai-Min Hui

**Affiliations:** 1 Fosun Pharma USA Inc., Boston, MA 02421, USA; 2 Shanghai Fosun Pharmaceutical Industrial Development, Co., Ltd., Shanghai 200233, China

**Keywords:** mRNA, vaccines, delivery route, formulations, delivery methods, immunity

## Abstract

mRNA vaccines have been revolutionary in combating the COVID-19 pandemic in the past two years. They have also become a versatile tool for the prevention of infectious diseases and treatment of cancers. For effective vaccination, mRNA formulation, delivery method and composition of the mRNA carrier play an important role. mRNA vaccines can be delivered using lipid nanoparticles, polymers, peptides or naked mRNA. The vaccine efficacy is influenced by the appropriate delivery materials, formulation methods and selection of a proper administration route. In addition, co-delivery of several mRNAs could also be beneficial and enhance immunity against various variants of an infectious pathogen or several pathogens altogether. Here, we review the recent progress in the delivery methods, modes of delivery and patentable mRNA vaccine technologies.

## 1. Introduction

Messenger RNA (mRNA) is a transient intermediate between DNA and proteins, which was first discovered in 1961 [1]. Later, mRNA was in vitro transcribed and emerged as a candidate for therapeutic purposes, including immunotherapies, viral vaccines, genome editing and cellular reprogramming [1]. The desired therapeutic effects of mRNA can be achieved only if the mRNA molecule reaches a specific target cell and produces enough protein of interest [2]. The methods and delivery of mRNA intracellular delivery present a major barrier to the broader application of mRNA therapeutics [3]. The intracellular delivery of mRNA is more challenging because of the larger size of mRNA (300–1500 kDa) as compared to a smaller size (4–14 kDa) for small interference RNA (siRNA) and antisense oligonucleotide (ASO) [4,5]. The major barrier to mRNA delivery to the target cell is the cell membrane reaching the cytoplasm. The cell membrane is composed of a zwitterionic lipid bilayer and negatively charged phospholipids [6,7]. The negatively charged mRNA molecules face repulsion from the cell membrane, which is also negatively charged. mRNA is also susceptible to degradation by ribonucleases in the extracellular environment. Therefore, the mRNA molecule needs protection from degradation by nucleases and shields its negative charge [8,9].

A variety of mRNA delivery methods have been developed, such as direct injection of naked mRNA, lipid-based carriers, polymers and protein derivatives [10]. Compared to other delivery vehicles, lipid nanoparticles have been extensively studied for the delivery of small molecules, such as siRNA and mRNA [6].

The utility of lipid nanoparticles to deliver mRNA has been successfully demonstrated with COVID-19 vaccines, such as mRNA-1273 and BNT162b [11]. More than 10% of vaccines being tested for use against SARS-CoV2 in humans are mRNA-based vaccines, including mRNA-1273, BNT162b, CVnCoV, ARCoV, ARCT-02, ChulaCov19 and LNP-nCoV saRNA. Of all the seven mRNA vaccines in clinical trials, only mRNA-1273 and BNT162b have been approved for use in humans. Other LNP-mRNA formulations have also been used for genetic diseases, virus infections and cancer [12,13].

Despite the recent advances in mRNA vaccines, there is growing interest in developing improved safety and effective delivery methods for mRNA-based therapies. With the emergence of the COVID-19 global pandemic, lipid-nanoparticle-based mRNA vaccines have emerged as the fastest and most efficient tool for combating such infectious diseases [14]. mRNA vaccine delivery is important to achieve vaccine efficiency. In this review, we summarize the mRNA delivery strategy, including the delivery barrier, the routes of administration and delivery vehicles that have been utilized both in preclinical and clinical settings. 

## 2. mRNA Therapeutic Delivery Barriers

mRNA-based vaccines have several benefits over other vaccines. mRNA vaccine sequences are precise and express a specific antigen leading to the induction of a directed immune response [15]. mRNA vaccines promote both humoral and cellular immune responses and induce the innate immune system. Additionally, nucleoside modification in the mRNA sequence reduces its inflammatory capacity. Therefore, mRNA-based vaccines are safe for delivery when compared to virus-based vaccines. The safety concerns for the viral contaminants are minimized since production is based on an in vitro cell-free transcription reaction [14].

In addition to the benefits of mRNA vaccines, there are several challenges associated with mRNA delivery that need to be addressed. The development of optimal delivery systems to protect mRNA and efficient delivery into the cells remains an area of active research [16]. mRNA vaccines might cause side effects, such as heart and renal failure, allergy and infarction [17]. mRNA vaccines may be degraded quickly in the body after administration or cause a cytokine storm, which poses a challenge to their delivery.

The delivery of mRNA into the cell is the most challenging part of mRNA therapeutics. The mRNA must cross several extracellular and intracellular barriers before it arrives in the cytoplasm or nucleus of its target cell [7,18].

The extracellular barriers include the cell membrane, which is composed of a lipid bilayer of a zwitterionic negatively charged phospholipid, ion pumps and channels, which maintain negative potential, thus, making it difficult for the mRNA to enter the cell [19]. Additional extracellular barriers include the presence of exonucleases or RNases in skin and blood. These exonucleases digest mRNA before its entry into the cell [7].

The intracellular barriers include endosomal escape, RNA sensors and endonucleases. Once the mRNA-based vaccine encounters the plasma membrane, it is engulfed and processed by an endocytic pathway to be released into the cell. However, a small percentage of LNPs evade the endocytic pathway due to the disruption of the endosomal membrane caused by the protonation of the LNP residual amines [10,20]. This leads to the premature release of LNP-mRNA cargo into the cell affecting the efficiency of the mRNA vaccine [21]. Another barrier to mRNA vaccines is the recognition of the mRNA by cytosolic innate sensors, such as toll-like receptors (TLRs), TLR 3 and TLR7. The TLRs bind to the mRNA and activate the inflammatory pathways, leading to the production of type I interferon and other inflammatory mediators, which results in inhibition of the cellular translation [22,23]. Additionally, intracellular RNases also act as a barrier by degradation of mRNA, even before it is translated to produce the antigen in the cell [24,25].

## 3. Routes of Administration

The route of mRNA vaccine delivery is essential to determine the efficacy of the vaccination. The anatomical and physiological properties of the site of vaccination, such as skin, lymphoid organ or muscle, affect the safety and efficacy of the vaccine [24,26]. The vaccines can be administered via either systemic or local applications [27,28,29].

**Figure 1:**
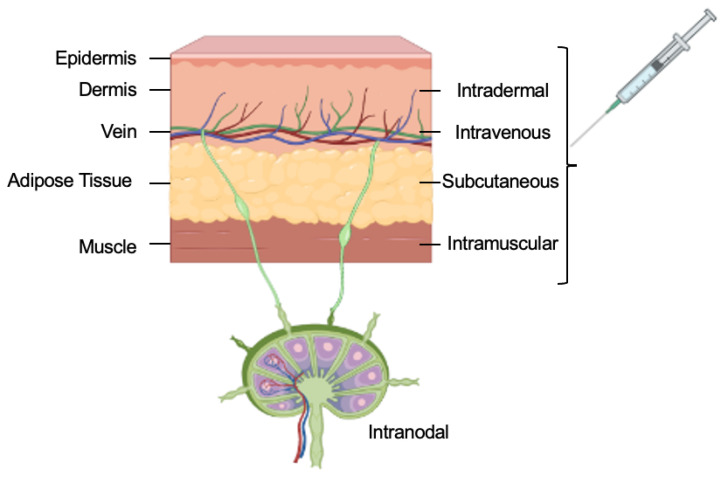
The routes of delivery for mRNA vaccines.

Systemic delivery is the delivery route where the vaccine is directly injected into the bloodstream to reach and affect all cells in the body [18,30], such as intravenous injections [31]. Local injections are the mode of delivery where the vaccine is directly administered at the site of action. This route of delivery reduces the risk of side effects associated with systemic delivery (Figure 1). There is also much interest in targeted delivery [32]. This mode of delivery aims at direct injection into the target tissue or the organ. The intranodal injection is such an example of targeted delivery.

### 3.1. Intravenous Injections

Intravenous (IV) injections deliver mRNA vaccines into the systemic circulation in the body. IV injection provides the largest volume among all the other routes of vaccine administration. It was also observed that the total amount of antigen produced by IV is the highest when compared to the other routes of vaccine delivery [2,10]. IV injections allow for the direct access of mRNA vaccines to the immune cells and lymphoid organs in the circulatory system, which then leads to increased efficacy of the mRNA vaccines [33]. Even though IV is an efficacious method, there are several disadvantages, which include hindrance to vaccine delivery in the bloodstream by plasma proteins, enzymes and mechanical forces [34]. In addition, the mRNA and its delivery carriers might introduce systemic side effects, including spleen injury and depletion of the lymphocytes [18].

### 3.2. Subcutaneous Injections

Subcutaneous (SC)-injection-based mRNA vaccines are administered under the epidermis and dermis in the subcutis layer of the skin [35]. This layer of skin constitutes a loose network of adipose tissues and few immune cells. The loose adipose tissue at the injection site allows for a larger injection volume, which lowers the pressure and reduces pain at the site [36]. The larger injection volume also allows for efficient draining of the immune system. However, one of the disadvantages of the SC injection is that the rate of absorption is low and unintended degradation of the mRNA may occur [36,37].

### 3.3. Intramuscular Injections

Intramuscular (IM) injections are the most used route of administration for mRNA vaccines. Muscles are composed of a large network of blood vessels that recruit and circulate various immune cells, such as antigen-presenting cells (APCs), to the site of the injection [38]. IM-injection-based vaccines are administered into muscles with deeper tissue under the dermal and subcutaneous layer of the skin [39]. It has been shown that the IM-administered LNP-mRNA remains at the site of injection and in the draining lymph nodes for ~28 h [37,40]. IM has been used for mRNA vaccines against the RBD of SARS-CoV-2 virus and has been successful in conferring immunity against the virus [41].

### 3.4. Intradermal Injections

Intradermal injections are administered in the dermis layer of the skin, a dense connective tissue, which has vascular and lymphatic vessels, which help transport the mRNA vaccines and APCs to the draining lymph nodes to activate B and T cells [42].

### 3.5. Intranodal Injections

Intranodal injections deliver the mRNA vaccines in the peripheral lymphoid organs where APCs and immune cells, such as B and T cells, interact. The APCs in the lymphoid organs readily engulf the mRNA vaccine [43]. There are reports on the efficacy of intranodal-based DNA, peptide and protein vaccines but its efficacy for mRNA vaccines remains to be explored [44]. Additionally, the administration of the intranodal mRNA vaccines needs the guidance of ultrasound for the administration [45].

Other delivery methods include intranasal injection or inhalation-based delivery of mRNA vaccines, which are studied in respiratory delivery [46,47,48]. The inhaled materials encounter pathogen-associated molecular patterns (PAMPs) and damage-associated molecular patterns (DAMPs), which derive APCs to take up the antigen. The dendritic cells then migrate via lymphatic vessels to the lymph nodes where the antigen is presented via MHC II complexes to naïve B and T cells. In addition, the respiratory system has inducible bronchus-associated lymphoid tissue (iBALT), which consists of B-cell follicles, plasma cells, T cells and APCs. Antigens are then presented to both the effector and naïve B and T cells. These tertiary lymphoid tissues are interconnected with the mucosal-associated lymphoid tissue (MALT) [47,49].

## 4. Delivery Vehicle

### 4.1. Naked mRNA

**Figure 2:**
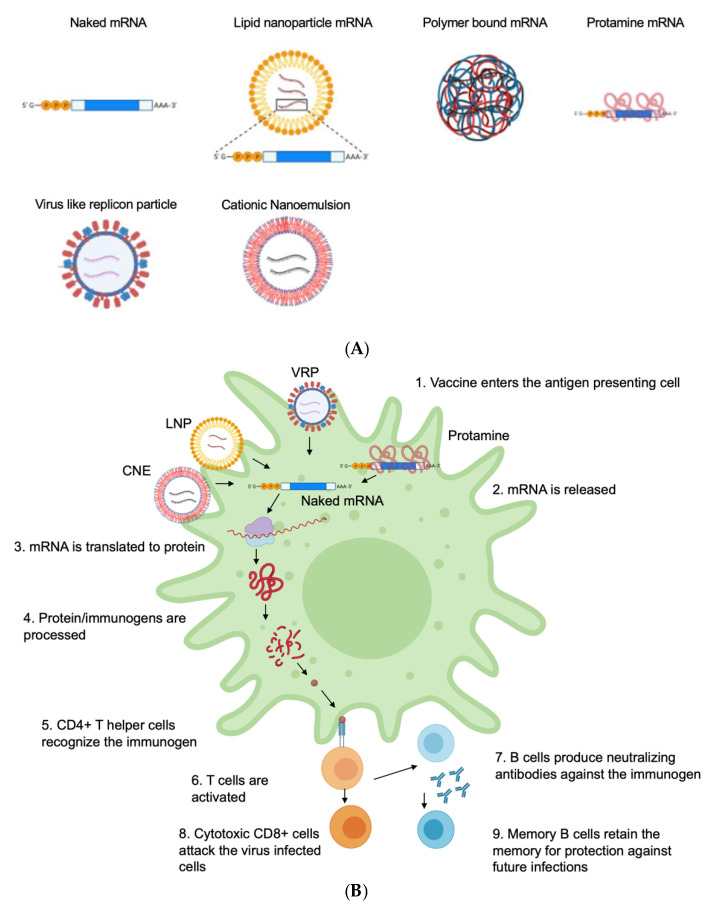
(**A**) Delivery carriers for mRNA vaccines and (**B**) the mechanism of mRNA delivery into the cell.

Naked-mRNA-based vaccines are delivered by dissolving the mRNA into a buffer and then injecting the mRNA solution directly into the body (Figure 2A) [24]. Although naked mRNA cannot diffuse across the intracellular membrane, the mechanism for its internalization remains unclear [50]. Initially, it was proposed to be internalized by a cellular process, known as micropinocytosis [51,52]. Some studies have suggested that the internalization of naked mRNA is facilitated by mechanical forces, such as hydrostatic pressure. It is speculated that hydrostatic pressure may lead to the disruption of the cell membrane and facilitate cytosolic delivery of nucleic acids [53]. Some of the benefits of naked mRNA-based vaccines include storage stability and intrinsic immunogenicity [54]. Naked mRNA can be easily stored at 4 °C for up to 10 months in 10% trehalose upon freeze drying [54]. Similarly, the intrinsic immunogenicity is advantageous because it triggers RNA sensors, such as TLRs, RIG-I, PKR and IFIT1, which leads to the activation of NF KB, type I IFN pathways and the release of cytokines. Some reports have also indicated that naked mRNA induces an innate immune response [55]. Unmodified naked mRNA is considered a strong stimulator of TLR3/7/8 and PKR, although mRNA translation might be inactivated by certain RNA sensors in the cytosol [56,57]. In addition, naked mRNA vaccines are also susceptible to RNase degradation and intracellular delivery [58,59]. These obstacles can be removed by local administration of mRNA, such as intramuscular, intranodal, intratracheal, intradermal and intranasal routes, to minimize their contact with RNases in the bloodstream [43,56,60,61,62,63,64,65]. Naked mRNAs have been tested in clinical trials against diseases, such as melanoma, influenza and HIV-1 virus (Table 1) [66,67].

### 4.2. Electroporation for Dendritic-Cell-Based mRNA Vaccines

Antigen-presenting cells (APCs) are responsible for the internalization, processing and presentation of antigens to lymphocytes. Dendritic cells (DCs) are types of APCs that present processed antigens from microorganisms, tumor cells and virus-infected cells to T cells for the generation of the immune response [68,69,70]. DCs are suitable vaccination targets because of their migration to T cells in the lymph nodes, high expression of MHC, co-stimulators and cytokines [71]. Electroporation disrupts the cell membrane by generating electric shock for intracellular nucleic acid delivery. Delivery efficiency can be improved by adjusting voltage, capacitance, resistance and other factors, such as cell number, density, RNA quantity and pulse time [53,72,73]. Electroporation has been used for DC-based mRNA vaccines in clinical trials [74,75,76].

### 4.3. Peptide-Based Delivery Protamine

Peptides have been used as delivery carriers for mRNA vaccines [77,78]. To serve as delivery carriers, the peptides should contain strings of positively charged amino acids, such as lysine and arginine. This allows for the formation of electrostatic interactions between positively charged peptides and negatively charged mRNA, thus, enabling a spontaneous complex formation [79,80,81]. Protamines are advantageous as carriers of mRNA vaccines because they protect the mRNA and make it less susceptible to being degraded by RNases [82,83]. The protamine–mRNA complex has high adjuvant activity. The complex is immunogenic via activation because of its structural similarity to viral RNA genome [84,85]. The feasibility of the mRNA protamine complex was tested with β-galactosidase mRNA—protamine—which was injected into a glioblastoma tumor. It was observed that the mRNA complexed with protamine was poorly translated [86,87].

### 4.4. Polymer-Based Delivery

Polymers are functional materials that can deliver mRNA vaccines. Like protamines, polymers protect mRNA from RNase degradation [3]. Polymer-based mRNA nanoparticles have high polydispersity and to stabilize this, formulation modifications, such as incorporating lipid chains, hyperbranched groups and biodegradable units, are being explored. Cationic polymers include polyethyleneimine (PEI), polyamidoamine (PAMAM) dendrimer and polysaccharides [88,89,90,91]. PEI has been a widely used polymeric material for mRNA delivery and can be prepared by directly mixing PEI with RNA solution. PEI formulation was used to deliver a HIV gp120 mRNA-based intranasal vaccine in mice [92,93]. In addition, PEI-based formulation was used to deliver HIV-1 Gag and Pol antigens to produce T-cell response against HIV infections upon intramuscular vaccination in mice [93]. This vaccination approach protected mice against the viral challenge. PEI formulation has also been used for the delivery of self-amplifying mRNA encoding hemagglutinin antigens from various influenza strains for immunization in mice models [90]. Even though PEI has shown in vivo efficacy, the possible toxicity has hindered its development [3].

Another polymer that has been utilized is polyamidoamine (PAMAM), which is a cationic polymer. The PAMAM dendrimer has been used to deliver intramuscular self-amplifying mRNA-based vaccination against Toxoplasma gondii, Ebola and H1N1 influenza virus [91,94].

Like PAMAM, Chitosan, which is a polysaccharide material, has been used to condense and deliver self-amplifying mRNAs encoding hemagglutinin and nucleoprotein for influenza virus [8].

Polymer materials for the delivery of mRNA vaccines have been proven to be efficient in preclinical studies [95]. However, new functional polymers, with improved biodegradability and delivery efficacy, are desirable before the translation of polymer-based mRNA vaccines in the clinic.

### 4.5. Lipid-Based Delivery

#### Lipid Nanoparticles (LNPs)

Lipid-derived nanoparticles are widely used for in vivo delivery of mRNA vaccines [10,21,24,96]. They are composed of nano-sized particulates that are composed of synthetic lipid materials. LNP-based mRNA vaccines encapsulate RNA and protect it from RNAse-mediated degradation (Figure 2A) [21,97]. In addition, LNPs deliver mRNA molecules effectively through endocytosis mechanisms (Figure 2B). LNPs are generally composed of a functional lipid component that is crucial for the intracellular RNA delivery [98,99,100]. The cationic or ionizable lipid materials, such as 1,2-di-O-octadecyl-3-trimethylammonium propane (DOTMA), N, N-Dimethyl-2,3-bis[(9Z,12Z)-octadeca-9,12-dienyloxy]propan-1-amine (DLinDMA) and N1, N3, N5 -tris(3-(didodecylamino)propyl)benzene-1,3,5-tricarboxamide (TT3), with one or multiple amino groups, can be positively charged at a certain pH, which helps encapsulate the negatively charged mRNA molecules via electrostatic interactions and associate with the cell membrane [26,101,102]. Further, the ionizable cationic lipids interact with the anionic endosome membrane, which leads to the formation of a disruptive non-bilayer structure, which leads to the release of the mRNA into the cytosol. It has been indicated that the hydrophilic head of the lipid material determines the acid dissolution constant (pKa) and influences the delivery efficiency of the LNPs [103]. Although the lipid materials enhance efficacy, they might have some side effects on the cells [104]. The polyethylene glycol (PEG) lipid conjugates stabilize the nanoparticles during preparation and prolong the circulation time in vivo, which leads to adverse effects, such as anaphylaxis, that can lead to rashes, shortness of breath and plummeting blood pressure [105,106,107,108]. Additionally, the cyclic amino head groups of LNPs bind directly to the stimulator of interferon gene (STING) protein and activate the downstream signaling pathway, which leads to enhanced immune response [109]. The delivery routes of mRNA vaccines may impact their efficacy by affecting the distribution pattern and the expression pattern of the encapsulated mRNA [21]. The local injections, such as intramuscular, intradermal and intranasal administration, lead to infiltration of antigen-presenting cells, which stimulates strong and prolonged local expression [21,110]. LNPs were used to deliver the COVID-19 mRNA vaccine. The TT3-LNP was used to deliver the receptor-binding domain of SARS-CoV-2 intramuscularly, which led to the expression of the antigen in the muscle tissue [26]. Overall, LNP-based mRNA vaccines have efficacy in preventing infectious diseases and providing immunity [111].

### 4.6. Cationic Nanoemulsion

Cationic nanoemulsion (CNE) utilizes nanoemulsion with cationic lipids for RNA delivery. Nanoemulsion has hydrophobic and hydrophilic surfactants to stabilize the oil core in the aqueous phase, thereby generating particles. Nanoemulsions are produced using methods, such as vigorous agitation, ultrasound and microfluidics [112]. One of the oils in water nanoemulsions approved by the FDA is MF59, which was used as an adjuvant with inactivated flu vaccine for the elderly. MF59 consists of squalene, sorbitan trioleate, polyoxyethylene, sorbitan monooleate and citrate buffer [113]. The efficacy of vaccines by MF59 is enhanced by the MyD88-mediated release of chemokines and recruitment of immune cells [114,115,116]. CNEs have been used for the delivery of mRNA vaccines against bacterial and viral infections [117,118,119,120]. Three chimeric MF59-CNE-based mRNA vaccines against the respiratory syncytial virus (RSV), HIV and human cytomegalovirus (CMV) were intramuscularly injected into mice, rabbits and rhesus macaques [117]. The vaccines induced high antigen-specific IgG titer and an efficient leukocyte infiltration [117]. Overall, CNE is efficacious as a delivery method in preclinical studies; however, its efficacy in clinical trials remains to be evaluated.

**Table 1:** mRNA vaccines in various animal models.

Delivery Method	Routes of Administration	Target	Immune Response	Animal Model	References
Naked mRNA	Intramuscular	Tumors	Humoral	Mice	[60]
	Intramuscular	Influenza, RSV, Encephalitis	Humoral	Mice	[61]
	Intradermal	Influenza	Humoral/Cellular	Mice, Human	[56]
	Intranodal	Influenza	Humoral	Mice	[43]
	Intranasal	Tuberculosis	Humoral	Mice	[65]
Dendrimer	Intramuscular	Ebola, Influenza and Toxoplasma	Humoral/Cellular	Mice	[91]
	Intramuscular	Zika	Humoral/Cellular	Mice	[121]
Protamine	Intradermal, Intramuscular	Rabies virus	Humoral	Mice, Pigs, and human	[82]
	Intradermal	Influenza	Humoral/Cellular	Mice, Ferrets, and pigs	[122]
	Intradermal	Prostate cancer	Cellular	Human	[123]
Polymer	Intramuscular	Influenza	Humoral	Mice	[124]
	Intranasal	HIV-gp120	Humoral	Mice	
	Intramuscular	HIV-Gag and Pol	Humoral/Cellular	Mice	[93]
	Subcutaneous	Zika	Humoral/Cellular	Mice, Rabbit	[90]
Lipid nanoparticle	Intradermal	Zika	Humoral	Mice, Primates	[12]
	Intramuscular	Ebola	Humoral	Guinea pigs	[125]
	Intradermal	HIV-Env	Humoral/Cellular	Mice	[126]
	Intravenous	HIV-IgG	Humoral	Mice	[127]
	Intramuscular	SARS-CoV2	Humoral	Human	[128,129]
Cationic nanoemulsion	Sub cutaneous	HIV—Gag	Humoral	Mice	[130]
	Intramuscular	RSV, CMV, HIV	Humoral	Mice, Rabbit, Macaques	[117]
	Intramuscular	Encephalitis	Humoral	Mice	[120]
Virus-like replicon particle	Intradermal	Dengue	Humoral	Macaques	[131]
	Intravenous, Intramuscular	Influenza	Humoral/Cellular	Mice, Swine	[132]
	Intramuscular	HIV	Humoral/Cellular	Mice	[133]
	Subcutaneous, Intramuscular	Ebola	Cellular	Primates	[134]
	Intramuscular	SARS-CoV	Humoral	Mice	[135]
	Intranasal	MERS-CoV	Humoral	Mice	[136]
	Intradermal	RSV	Humoral	Primates	[137]
	Subcutaneous, intramuscular	CMV	Humoral	Human	[138]

### 4.7. Virus-Like Replicon Particle (VRP)

Viral particles can be used to package and deliver antigen-encoding self-amplifying mRNA in cytoplasm like a virus [139]. Self-amplifying mRNA can then replicate and efficiently express the designated antigens. VRPs are efficient in cytoplasmic delivery of RNA payload by viral vectors [92,140]. This is because viruses internalize and release their genomes into cells via different pathways with high efficiency (Figure 2B) [141,142]. The most used VRPs for vaccines are single-stranded RNAs, including alphavirus, flavivirus, rhabdovirus and measles virus [139]. VRP vaccines were injected intradermally in non-human primates to produce immunity against the Venezuelan equine encephalitis virus (VEEV) [131]. Similarly, a Kunjin virus-derived VRP expressing GM-CSF was injected intratumorally in mice with colon carcinoma, which led to complete removal of the primary tumor and a reduction in lung metastases [143]. However, there are challenges for VRP-based mRNA vaccines, including the process of generating VRPs, which limits scaling up in the production of VRPs [144,145]. Another challenge is that, sometimes, there is antibody generation against viral vectors as well, which hinders the human clinical trials of VRP-based HIV-1 vaccines [146,147].

The use of an optimal delivery carrier for mRNA vaccine entry into the cell is essential for the efficacy of the vaccines. Naked mRNA is prone to degradation and, hence, offers limited efficacy in vivo. However, lipid-based nanoparticles have proven to be successful to deliver mRNA vaccines in humans, as seen in the case of mRNA-1273 and BNT162b as well as remain in clinical trials against various infectious diseases (Table 2). LNPs protect the mRNA from the RNases and help in the effective delivery of mRNA-based vaccines. However, further studies are required to explore the efficacy of other delivery methods, such as CNE, protamine, and VRP in the clinic.

## 5. Future Perspectives

mRNA vaccine delivery has been effective in preclinical studies and clinical trials; however, there are some challenges that need to be addressed. One such challenge is that during the process of delivery, a large proportion of RNA-loaded carriers becomes trapped in the endosome and gets degraded, thus, decreasing the efficacy [153,154]. Developments that enhance endosomal escape and prevent degradation are highly desirable. Another challenge is targeting the delivery to a specific site in vivo. The current delivery methods induce a plethora of immune cells at the site of injection, which leads to immune stimulation [155,156]. In vivo targeting of B cells, T cells, macrophages and dendritic cells will aid in increasing immunization efficiency [156]. The safety of the delivery vehicles, such as polymers and cationic lipids, remains a concern. These delivery vehicles may induce enhanced membrane fusion, endosomal disruption and cell stress, which can lead to cytotoxicity [157,158]. Therefore, safe delivery materials, such as biodegradable materials or the ones that mask cationic charges, are necessary.

The molecular mechanisms in the delivery process remain to be explored in depth to facilitate the development of effective immunization by mRNA vaccines. A better understanding of the delivery formats, administration routes and carrier materials, as well as pathways responsible for cellular uptake, cytosolic release, endosomal escape, lysosomal degradation and exocytosis is also required.

Given the success of mRNA vaccines in the COVID-19 pandemic, they are a promising alternative to the traditional vaccine platforms. mRNA vaccines are manufactured quickly and designed for emerging infectious diseases [159]. Henceforth, there has been an increase in the intellectual property (IP) landscape for mRNA-based vaccines. A recent report generated a ten-year landscape for mRNA vaccines’ IP. They identified 113 INPADOC patent families and indexed them based on the indication, methods of delivery and pharmacological modifications. It was also observed that patent filing dramatically increased over the past 5 years for cancer and infectious diseases. There were increased patent applications for emerging infectious diseases, such as Ebola virus, Zika virus, MERS-CoV2 and SARS-CoV2. Around 70% of the patents were filed by industry and the remaining were filed by academic institutions or independent investors. There was an increase in patent filing to protect the methods to improve mRNA delivery efficiency, especially for lipid-based nanoparticles, followed by nucleoside modified, sequence or codon-optimized mRNA or poly-A tail modified and self-amplifying mRNA [160].

During the COVID-19 pandemic, LNP-based mRNA vaccines have proven to be a quick and effective vaccination strategy; several other mRNA vaccines against various infectious diseases, such as HIV, Rabies virus, Influenza virus, Zika virus, Ebola virus and cancers, remain in clinical trials. However, there is a need to optimize the safety and increase the efficacy of mRNA vaccines [26]. So far, LNPs have been proven to be an effective delivery method for vaccination against SARS-CoV2 in humans [8]. The improvements in delivery methods and vaccine formulations will make mRNA vaccines an important class of vaccines against emerging infectious diseases and cancers.

## Figures and Tables

**Table 2:** mRNA vaccines against infectious diseases in clinic.

Disease	Vaccine Name	Company	Route of Administration	Phase	Platform	Reference
	mRNA-1273	Moderna	IM	Approved	LNP	[148]
	BNT162b	Pfizer/BioNTech/Fosun Pharma	IM	Approved	LNP	[129]
	CVnCoV	CureVac	IM	IIb/III	LNP	NCT04652102 [149]
SARS-CoV2	LUNAR-COV19	Arcturus Tx	IM	II	LNP	NCT04668339
	LNP-nCoVsaRNA	Imperial College London/VacEquity Global Health	IM	I	LNP	NCT04934111
	ARCoV	Academy of Military Science/Walvax Biotech/Suzhou	IM	III	LNP	NCT04847102
	ChulaCoV19	Chulalongkorn University	IM	II	LNP	NCT04566276
Rabies	CV7201	CureVac	IM	I	LNP	[150]
	CV7202	CureVac	IM	I	LNP	[151]
Influenza	mRNA-H10N8, mRNA-H7N9	Moderna	IM	I	LNP	[152]
Respiratory syncytial virus (RSV)	mRNA-1345	Moderna	IM	I	LNP	NCT04528719
Human metapneumovirus (HMPV) and parainfluenza virus type 3 (PIV3)	mRNA-1653	Moderna	IM	Ib	LNP	NCT04144348 NCT03392389
Human Cytomegalovirus (HCMV)	mRNA-1647	Moderna	IM	III	LNP	NCT05085366
Zika virus	mRNA-1893	Moderna	IM	II	LNP	NCT04917861
